# Readthrough acetylcholinesterase (AChE-R) and regulated necrosis: pharmacological targets for the regulation of ovarian functions?

**DOI:** 10.1038/cddis.2015.51

**Published:** 2015-03-12

**Authors:** J Blohberger, L Kunz, D Einwang, U Berg, D Berg, S R Ojeda, G A Dissen, T Fröhlich, G J Arnold, H Soreq, H Lara, A Mayerhofer

**Affiliations:** 1 Anatomy III – Cell Biology, Ludwig-Maximilian-University (LMU), Munich, Germany; 2 Neurobiology, Department Biology II, Ludwig-Maximilian-University (LMU), Munich, Germany; 3 ART, Bogenhausen, Munich, Germany; 4 Oregon National Primate Research Center/OHSU, Beaverton, OR, USA; 5 Laboratory for Functional Genome Analysis LAFUGA, Gene Center, Ludwig-Maximilian-University (LMU), Munich, Germany; 6 Department of Biological Chemistry and the Edmond and Lily Safra Center of Brain Science, The Hebrew University of Jerusalem, The Edmond J. Safra Campus, Givat Ram, Jerusalem, Israel; 7 Laboratorio de Neurobioquimica, Facultad de Ciencias Quimicas y Farmaceuticas, Universidad de Chile, Santiago, Chile

## Abstract

Proliferation, differentiation and death of ovarian cells ensure orderly functioning of the female gonad during the reproductive phase, which ultimately ends with menopause in women. These processes are regulated by several mechanisms, including local signaling via neurotransmitters. Previous studies showed that ovarian non-neuronal endocrine cells produce acetylcholine (ACh), which likely acts as a trophic factor within the ovarian follicle and the corpus luteum via muscarinic ACh receptors. How its actions are restricted was unknown. We identified enzymatically active acetylcholinesterase (AChE) in human ovarian follicular fluid as a product of human granulosa cells. AChE breaks down ACh and thereby attenuates its trophic functions. Blockage of AChE by huperzine A increased the trophic actions as seen in granulosa cells studies. Among ovarian AChE variants, the readthrough isoform AChE-R was identified, which has further, non-enzymatic roles. AChE-R was found in follicular fluid, granulosa and theca cells, as well as luteal cells, implying that such functions occur *in vivo*. A synthetic AChE-R peptide (ARP) was used to explore such actions and induced in primary, cultured human granulosa cells a caspase-independent form of cell death with a distinct balloon-like morphology and the release of lactate dehydrogenase. The RIPK1 inhibitor necrostatin-1 and the MLKL-blocker necrosulfonamide significantly reduced this form of cell death. Thus a novel non-enzymatic function of AChE-R is to stimulate RIPK1/MLKL-dependent regulated necrosis (necroptosis). The latter complements a cholinergic system in the ovary, which determines life and death of ovarian cells. Necroptosis likely occurs in the primate ovary, as granulosa and luteal cells were immunopositive for phospho-MLKL, and hence necroptosis may contribute to follicular atresia and luteolysis. The results suggest that interference with the enzymatic activities of AChE and/or interference with necroptosis may be novel approaches to influence ovarian functions.

Necrosis was for a long time considered to be an unregulated form of cell death that is beyond control. Apoptosis, by contrast, was considered the only form of regulated cell death that has roles not only in tissue homeostasis but also in development and disease. This view has changed and numerous morphologically and biochemically distinct forms of cell death have been recognized by now.^1, 2^ Programmed necrosis, termed necroptosis, participates in a growing number of diseases, for instance, ischemic injury in myocardial infarction and stroke, ischemia–reperfusion injury, atherosclerosis, inflammatory bowel diseases, pancreatitis, neurodegeneration, viral infection and other diseases.^1, 3, 4, 5, 6, 7^ Necroptosis can be initiated by death receptors (e.g., tumor necrosis factor (TNF) receptor 1 or Toll-like receptors (TLR)) by interferon receptor signaling and activation of the RNA-responsive protein kinase PKR, the DNA receptor DAI (DNA-dependent activator of interferon regulatory factors), as well as by other unknown means.^5, 8, 9, 10^ It requires the kinase activity of receptor-interacting serine/threonine-protein kinase 1 and 3 (RIPK1/3) and the formation of the necrosome. It involves among others a pseudokinase (MLKL; mixed lineage kinase domain like), which is phosphorylated by RIPK3 and then executes programmed necrosis. A recent report showed that in drug-induced liver injury the phosphorylated MLKL (p-MLKL) can be traced by a monoclonal antibody^11^ in human tissue. The execution involves the active integration of membranes (e.g., of mitochondria, lysosomes) and a balloon-like swelling of the cells.^12^ Importantly, necroptosis can be blocked by necrostatin-1 (Nec-1) or derivatives, and the MLKL inhibitor necrosulfonamide (NSA). This may be a clinically relevant approach to block cellular damage in diseases.^5^ Whether necroptosis has a role in physiological events is not well established.

In the ovary, follicles grow, and then eventually reach the ovulatory stage, in which the oocyte is released, or they undergo atresia, a process that involves cell death of granulosa cells (GCs) and of oocytes. Atresia is much more frequent and indeed is the main fate of ovarian follicles. It eventually results in the depletion of the ovarian pool of follicles, and in human this inevitably leads to menopause.^13, 14^ In small follicles of mice, there is evidence for not yet identified non-apoptotic mechanisms, which are likely responsible for atresia of primordial follicles.^15^ The ovarian cell death processes of larger follicles, as far as known to date, can involve apoptosis, autophagy, cornification and necrosis.^16, 17, 18, 19, 20, 21^ The morphological description of necrotic GCs, together with immune cells, that is, macrophages in ovarian follicles,^17^ is strong evidence for a necrotic process, possibly necroptosis, to occur.^1, 3, 4, 5, 6, 7, 22^

In the ovary, systemic and local signaling factors, that is, hormones and growth factors, influence the fate of follicles.^13, 23^ Neurotransmitters may have a say, as well.^24, 25^ Acetylcholine (ACh) stands out because it is synthesized in the ovary by non-neuronal GCs, which form the main cellular components of the follicle. The ovarian cholinergic system, which includes muscarinic receptors, is part of a widespread system of non-neuronal ACh production and local ACh actions. These are found throughout the body and are best examined in the immune system.^26, 27, 28^ The significance of the ovarian cholinergic system is not clear yet. Previous studies identified trophic, growth-promoting influences of ACh on GCs via muscarinic receptors in bovine and human ovary and derived cells.^29^ They include changes in the levels of intracellular Ca^2+^, increased levels of a master transcription factor (early growth response protein 1) and the activation of several ion channels.^30, 31, 32, 33, 34^ Given an important trophic role of ACh, its actions should be restricted spatially and temporarily. Two esterases cleave and inactivate ACh, butyrylcholinesterase (BChE) and acetylcholinesterase (AChE). BChE was described in proteomic studies as a component of follicular fluid (FF),^35, 36^ but little is known about ovarian AChE. Yet, histochemical data suggested the expression by GCs in some species.^37^ Several splice variants of AChE are known (e.g., AChE-E, -S, -R), which result in forms that differ in solubility and subcellular localization.^38, 39^ AChE-R furthermore exhibits a unique C-terminal sequence, which is responsible for non-enzymatic actions of this variant. It is involved in proliferation, apoptosis and development of various cell types in different organs, for example, brain and hematopoietic cells.^40, 41, 42, 43, 44, 45, 46^

To understand local regulation of ovarian functions by the cholinergic system, we studied expression and functions of AChE and BChE in human and in non-human primate ovary and derived GCs. We found that AChE-R is an ovarian factor, which can induce regulated necrosis (necroptosis) of GCs. We also found that the cholinergic system and necroptosis of GCs can be influenced pharmacologically and may represent novel drug targets allowing intervention with basic ovarian processes, namely proliferation, differentiation and cell death.

## Results

### Trophic ACh actions in human GCs are mediated by muscarinic receptors and are limited by intrinsic AChE

**Figure 1:**
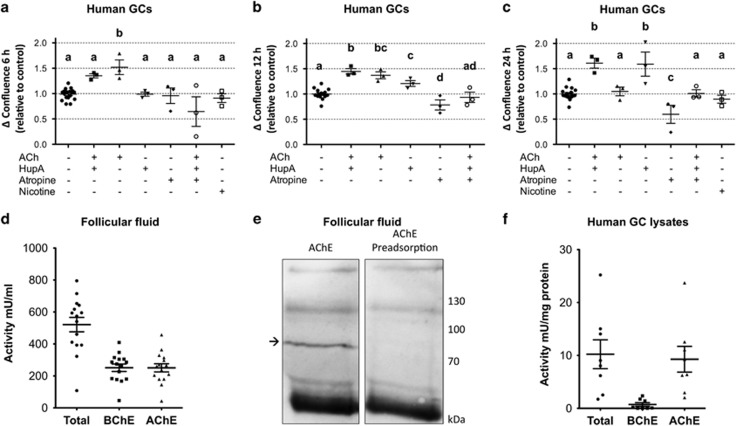
Monitoring of the ACh system in human GCs and evidence for AChE in FF and cultured GCs. (**a**–**c**) Change in confluence (relative to control) of cultured human GCs after 6, 12 and 24 h of treatment with different combinations of ACh (10 *μ*M), HupA (10 *μ*M), atropine (1 *μ*M) and nicotine (10 *μ*M). ACh initially shows a trophic effect on GCs but is degraded during 24 h of stimulation. HupA blocks ACh-degradation and the trophic ACh-effect remains visible after 24 h. Atropine is able to block the ACh-mediated effect and also decreases basal confluence change after 24 h. Nicotine was used as a control at 6 h and 24 h and shows no significant effect. Values are the mean±S.E.M. of *n*=3 independent preparations of cells pooled from two to five patients each. For each stimulation, a parallel control experiment was performed. Different letters indicate significant differences (*P*<0.05; analysis of variance). (**d**) AChE and BChE activity in FF, shown by the Ellman assay. Values are the mean±S.E.M. of *n*=15 FFs of different patients (Total: 521±45 mU/ml; BChE: 251±23 mU/ml; AChE: 251±26 mU/ml). (**e**) Identification of AChE protein in FF and a preadsorption western blotting experiment. Arrow indicates expected mass of protein (82 kDa). (**f**) Lysates of cultured human GCs possess AChE activity and low BChE activity in the Ellman assay. Values are the mean±S.E.M. of *n*=8 independent preparations of cells from two to five patients each (Total: 10±3 mU/mg; BChE: 0.7±0.3 mU/mg; AChE: 9±2 mU/mg)

To study functional components of the ovarian ACh system, we monitored cell confluence of cultured human GCs, that is, the major cell population of large human ovarian follicles. An increase in confluence due to cell spreading and/or cell number was regarded as a trophic influence. Several independent experiments showed a significant increase in confluence after addition of ACh (10 *μ*M; Figures 1a and b). After 24 h, no difference between ACh-treated and the control group was observed (Figure 1c), possibly because ACh becomes degraded. Indeed, when the AChE inhibitor huperzine A (HupA; 10 *μ*M) was added to ACh, a significant increase of confluence resulted after 12 and 24 h. This suggests the presence of an intrinsic ACh degradation system in the cultures, which can be blocked. AChE may be produced by GCs or be present in the medium, which was supplemented with fetal calf serum (FCS). Supporting the prediction, the addition of HupA alone also increased confluence, as seen after 12 h and 24 h and suggested that endogenous ACh production and action are amenable for manipulation. Blockage of the muscarinic ACh receptors of GCs by atropine (1 *μ*M) decreased confluence after 12 and 24 h. Simultaneous addition of ACh, HupA and atropine resulted in unchanged confluence levels compared with controls after 12 and 24 h. This indicates that the trophic ACh effect relies on the activation of muscarinic receptors. Nicotine (10 *μ*M) was not able to induce trophic effects in GCs. Thus ACh exerts trophic actions via muscarinic receptors in human GCs. The use of HupA revealed that AChE normally restricts trophic ACh actions. HupA imbalances the cholinergic system of production and breakdown events, and this action results in a net trophic action of ACh.

### Cholinesterase activity in FF and human GC lysates

Both AChE and BChE activity were detected in FFs from 15 *in vitro* fertilization patients (Figure 1d). AChE and BChE activities accounted for nearly the same amounts of activity. Western blotting revealed genuine AChE protein in FF (Figure 1e). The western blotting was repeated with FFs stemming from four different patients. Using an antibody against AChE, we yielded a band of the expected 82-kDa size. When the antibody was preadsorbed with the corresponding blocking peptide, the band disappeared. In lysates of cultured GCs, AChE activity was detected, whereas BChE activity was very low (Figure 1f). The results indicate that AChE is produced by human GCs, whereas BChE in FF may mainly be derived from the circulation.

### AChE isoforms in cultured human GCs

**Figure 2:**
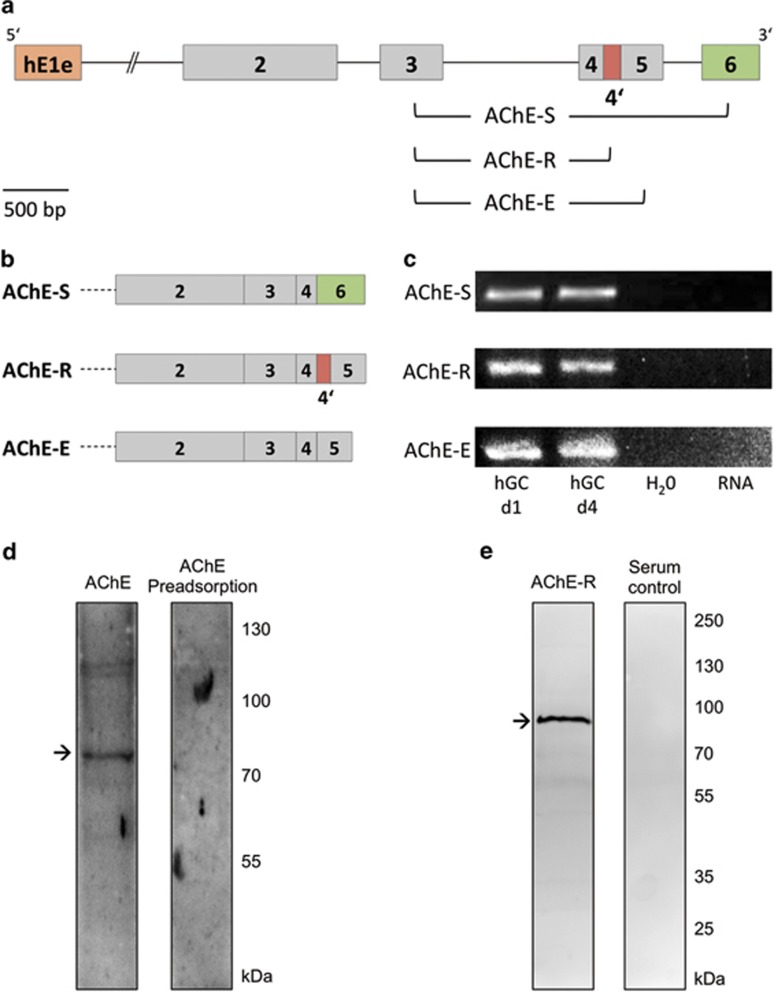
AChE variants in human GCs. (**a**) Simplified AChE gene structure with brackets indicating the position of PCR products. (**b**) Three possible 3′-AChE splice variants AChE-S, AChE-R and AChE-E. (**c**) RT-PCR and sequencing showed that the AChE-S, AChE-R and AChE-E variant are present in human GCs (hGC) at different days in culture. Water and RNA controls were negative. (**d**) AChE protein was detectable in human GCs by using western blotting. Arrow indicates the expected size of protein (82 kDa). (**e**) Western blotting with an antiserum specific for the AChE-R variant revealed the presence of this variant in human GCs. Arrow indicates the position of specific protein band. A serum control was negative

Reverse transcription-PCR (RT-PCR) strategies followed by sequencing allowed us to identify three AChE splice variants in human GCs: the readthrough (R), erythrocyte (E) and synaptic (S) AChE variant (Figures 2a–c). They were identified in GCs at different days of culture in six experiments with independent GC preparations. AChE protein was detected in GC lysates as well (four independent GC preparations). An antiserum recognizing all AChE variants and an antiserum specific for the R-variant were used for western blotting studies. The antiserum against all AChE variants revealed a band at the expected 82-kDa and this staining was not observed upon preadsorption with AChE (Figure 2d; two independent GC preparations). AChE-R protein was detected as a single band (Figure 2e; six independent GC preparations). Control blots in which the specific antisera were omitted also revealed the specificity of the results.

### Expression of AChE isoforms in non-human primate and human ovarian tissue

**Figure 3:**
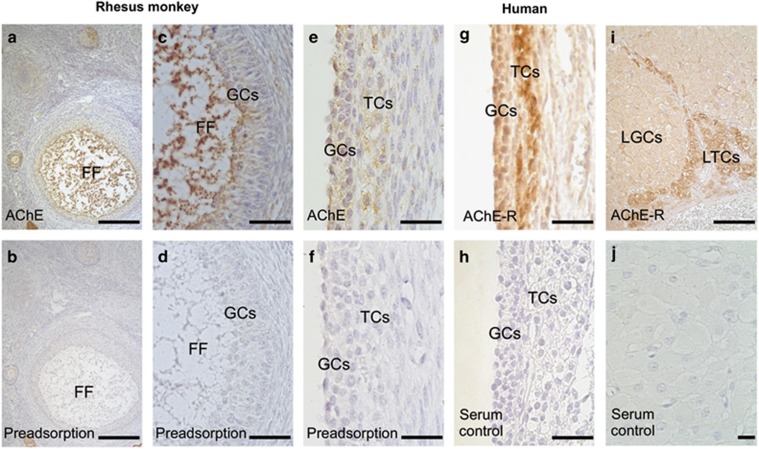
AChE and the AChE-R variant in ovarian tissue. (**a** and **c**) In rhesus monkey, ovarian tissue FF and GCs are positive for AChE in an immunohistochemical staining. (**b** and **d**) Preadsorption controls are nearly devoid of staining. (**e**) Immunohistochemistry using human ovarian sections shows positive staining for AChE in GCs and TCs of antral follicles. (**f**) Preadsorption control shows no staining. (**g**) GCs and TCs in human antral follicles are positive for the AChE-R variant. (**h**) Serum control lacks staining. (**i**) Human luteinzed GCs (LGCs) and luteinized TCs (LTCs) are positive for AChE-R. (**j**) Serum control lacks staining. Bars indicate 500 *μ*m (**a** and **b**), 80 *μ*m (**c** and **d**), 50 *μ*m (**e**–**h**), 100 *μ*m (**i**) and 50 *μ*m (**j**)

Immunohistochemical staining of rhesus monkey ovarian sections with an antiserum against all AChE variants revealed positive staining in FF and GCs of preantral and antral follicles (Figures 3a and c). In preadsorption experiments, this staining almost completely disappeared (Figures 3b and d). In human ovarian tissue, GCs and theca cells (TCs) of antral follicles were immuno-reactive for AChE and preadsorption confirmed staining specificity (Figures 3e and f). The AChE-R variant was identified in GCs and TCs of human antral follicles by using an antibody specific for this variant (Figure 3g). TCs showed stronger staining for AChE-R than GCs. No staining was found in the control experiment with serum only (Figure 3h). In addition to follicles, cells of the human corpus luteum specifically stained for AChE-R (Figure 3i). The staining of theca–luteal cells was more intense than the staining of granulosa–luteal cells and was not observed in control experiments (using serum instead of the antiserum; Figure 3j).

### The AChE-R synthetic peptide ARP increases cell death in cultured GCs

**Figure 4:**
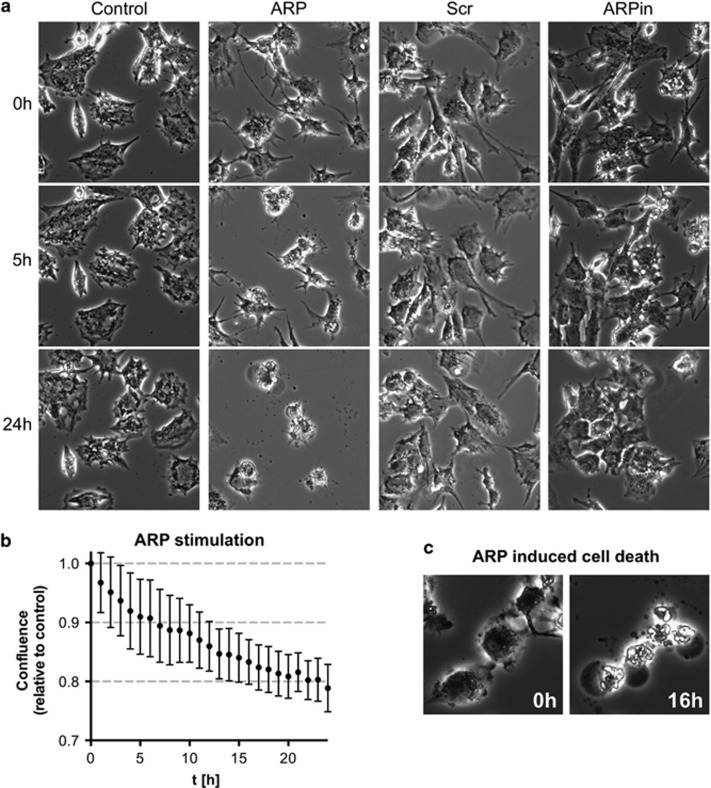
ARP increases cell death in cultured human GCs. (**a**) Live cell imaging details of GCs treated with ARP (50 ng/ml), Scr (50 ng/ml) and heat-inactivated ARP (ARPin; 50 ng/ml). Note decreased number of cells after 24 h ARP treatment. (**b**) ARP (50 ng/ml) stimulation decreases confluence in human GCs compared with control group during 24 h. Values are the mean±S.E.M. of *n*=3 independent preparations of cells from two to five patients each. (**c**) Characteristic morphology of ARP induced cell death at 0 and 16 h after ARP stimulation

**Figure 5:**
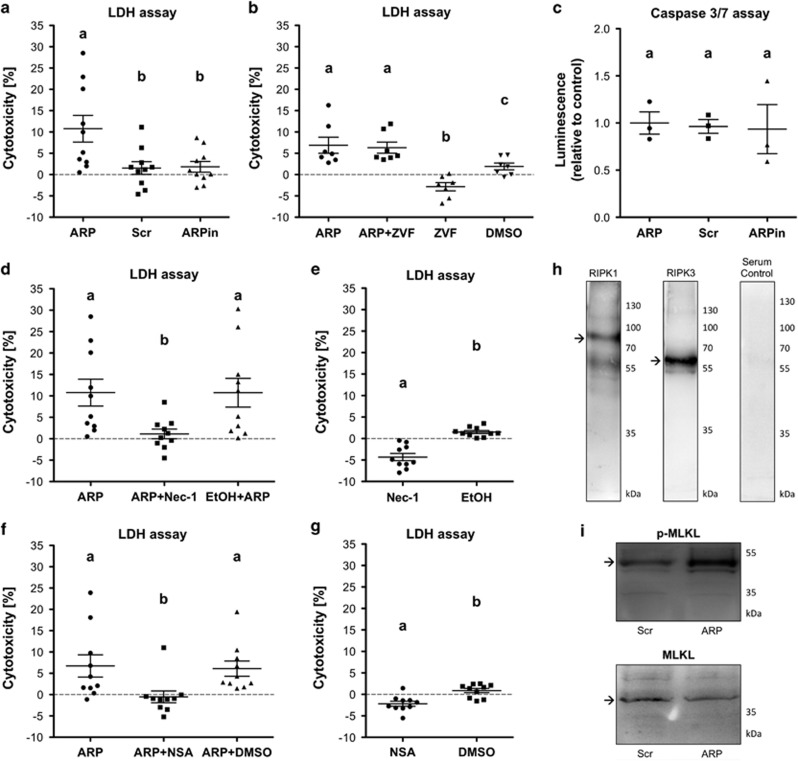
Nec-1 and NSA block ARP-induced increase in cell death, while Z-VAD-FMK does not. (**a**) LDH cytotoxicity assay performed with cultured human GCs. ARP (50 ng/ml) significantly increases cytotoxicity compared with control groups (Scr 50 ng/ml; ARPin 50 ng/ml; *P*<0.05; analysis of variance (ANOVA)). (**b**) Z-VAD-FMK (ZVF; 20 *μ*M) does not block ARP-regulated increase in cytotoxicity. Treatment with ZVF only significantly decreases cytotoxicity compared with control (DMSO 1‰ *P*<0.05; ANOVA). (**c**) No increased activity of caspase 3/7 was detected in ARP-treated cells compared with control groups (*P*<0.05; ANOVA). Values are the mean±S.E.M. of *n*=3 independent preparations of cells pooled from two to five patients each. (**d**) Nec-1 (20 *μ*M) significantly blocks ARP-regulated increase in cytotoxitcity. Ethanol (EtOH; 0.1‰) has no effect on ARP-dependent increase in cytotoxicity. (**e**) Treatment with Nec-1 causes significant lower cytotoxicity compared with control group (*P*<0.05, *t*-test). (**f**) NSA (0.5 *μ*M) is able to block ARP-dependent increase in cytotoxicity (*P*<0.05; *t*-test). DMSO (0.1‰) has no effect on ARP-dependent increase in cytotoxicity. (**g**) Treatment with NSA causes significant lower cytotoxicity compared with control group (*P*<0.05, *t*-test). All values of LDH-assays are shown as mean±S.E.M. of *n*=10 (except Z-VAD-FMK stimulation, panel **b**, *n*=7) independent preparations of cells from two to five patients each. (**h**) Identification of RIPK1 and RIPK3 protein in cultured human GCs by western blotting. Arrows indicate the expected mass of protein (RIPK1: 76 kDa; RIPK3: 57 kDa; p-MLKL: 54 kDa). Serum control was negative. (**i**) Identification of MLKL and p-MLKL in cultured human GCs by western blotting. ARP treatment for 5 h increased the levels of p-MLKL compared with control group, which was treated with the control peptide (Scr). Arrows point to the expected mass of the proteins (MLKL 37 kDa; p-MLKL: 54 kDa). Different letters in **a**–**g** indicate statistically significant differences between the treatment groups

In contrast to the AChE-S and AChE-E, the AChE-R is a soluble monomer and its specific C-terminal peptide ARP has been shown to possess additional non-enzymatic functions.^41^ To explore assumed non-enzymatic effects in human GCs, we used a synthetic ARP peptide (Figure 4). Live cell imaging performed over a 24-h time period revealed massive cell death events in the ARP-treated cells (50 ng/ml) compared with the untreated control group (Figure 4a; Supplementary Data). A scrambled control peptide (Scr; 50 ng/ml) and heat-inactivated ARP (ARPin; 50 ng/ml; 10 min, 95 °C) exhibited no bioactivity. Confluence measurements furthermore underpinned this observation (Figure 4b). Cell death events were first observed after approximately 2–3 h upon the addition of ARP and continued throughout a 24-h period. Many of the dying cells showed a characteristic morphology upon ARP treatment. It involved cytoplasmic ballooning (Figure 4c), which albeit at much lower frequency could be found in control cells as well. Lactate dehydrogenase (LDH) assays with 10 independent preparations of cultured GCs were performed to detect LDH release as measure for plasma membrane damage. The results showed a significant increase after 5 h, indicating cytotoxicity of ARP treatment compared with the control, Scr and ARPin (Figure 5a). The pan-caspase inhibitor Z-VAD-FMK (20 *μ*M) did not prevent the ARP-dependent increase in cytotoxicity seen in LDH measurements. Addition of Z-VAD-FMK to GCs, however, blocked basally occurring cell death, presumably apoptosis, which was observed in the control group, in which the solvent dimethyl sulfoxide (DMSO; 1‰) was tested (Figure 5b; seven independent GC preparations). ARP stimulation did not change the activities of caspase 3/7 over control groups (Figure 5c; three independent GC preparations). The results indicate that the type of cell death that is involved is not typical caspase-dependent apoptosis. The RIPK1 inhibitor Nec-1 (20 *μ*M) significantly blocked the ARP-dependent increase in LDH release when added to ARP-exposed GCs (Figure 5d; 10 independent GC preparations). This effect points to necroptosis as cause for the increased number of cell deaths by ARP stimulation. In the GC group treated with Nec-1 alone, a significantly reduced cytotoxicity became apparent, indicating a basal level of necroptosis in GCs (Figure 5e; 10 independent GC preparations). Addition of NSA (0.5 *μ*M), a blocker of MLKL, effectively reduced necroptotic cell death, indicated by its ability to inhibit ARP-induced cytotoxicity (Figure 5f; 10 independent GC preparations). As in the case of Nec-1, NSA significantly reduced cytotoxicity in GCs compared with untreated control (Figure 5g), and this further indicates that necroptosis is a form of cell death in GCs. As Nec-1 is dissolved in ethanol and NSA in DMSO, we excluded non-specific possible cytotoxic actions of the corresponding solvents (ethanol (0.1‰) and DMSO (0.1‰)) using the LDH assay, (Figures 5d and f). Furthermore, RIPK1, RIPK3 and MLKL, key proteins in the necroptosis pathway, were identified in three GC preparations by using western blotting (Figures 5h and i; for RT-PCR data, see Supplementary Data). ARP, but not the control peptide, increased the levels of p-MLKL after 5 h, which corresponds to the time, when the cytotoxicity of ARP was confirmed by LDH measurements (Figure 5i). This experiment was repeated using three independent GC preparations (Supplementary Data).

### Detection of p-MLKL in non-human primate and human ovarian tissue

**Figure 6:**
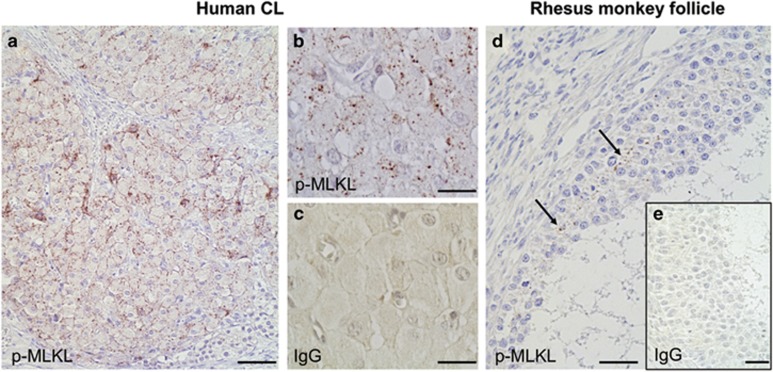
p-MLKL in primate ovarian tissue. (**a** and **b**) Cells in human corpus luteum (CL) are positive for p-MLKL. (**c**) IgG control lacks staining. (**d**) GCs in antral follicles of rhesus monkeys show positive staining for p-MLKL. (**e**) IgG control lacks staining. Bars indicate 100 *μ*m (**a**) and 40 *μ*m (**b**–**e**)

We used a monoclonal antibody, which was recently described^11^ and allows in human tissue immunohistochemical identification of p-MLKL, that is, a marker for necroptosis. The human corpus luteum showed specific staining for p-MLKL (Figures 6a and b), while the immunoglobulin G (IgG) control was negative (Figure 6c). In rhesus monkey follicles, the GCs were immunoreactive for p-MLKL (Figure 6d). The IgG control was devoid of staining (Figure 6e).

## Discussion

Several stimuli are known to initiate regulated necrotic cell death,^1, 5, 10^ but to our knowledge, AChE-R has not been described to be among them. AChE-R is one of the several splice variants of AChE, which is responsible for enzymatic ACh degradation. The results obtained in our study reveal that via additional non-enzymatic mechanisms it induces RIPK1-/MLKL-dependent regulated necrosis (necroptosis), that is, a hitherto unrecognized form of cell death in primary human ovarian cells.

AChE was previously linked to cell death.^47, 48^ It appeared, for example, to be involved in TNFalpha–induced apoptosis in Hela cells, endothelial cell lines and primary rat aorta smooth muscle cells. The prevalent isoform of AChE, expressed by apoptotic cells, was AChE-S and pharmacological inhibition of AChE prevented apoptosis.^49^ In neurons, an N-terminally extended N-AChE-S variant was identified as being apoptogenic.^50^ However, the splice variant AChE-R increased apoptosis of male germ cells and spermatogenesis.^51^ Apoptosis was defined mainly by positive TUNEL staining, which however may not be specific for apoptosis. The mechanism(s) by which AChE-R impaired spermatogenesis was thought to be related to, or mediated by, the known AChE-R interaction partner, the scaffold protein RACK1 (receptor of activated C kinase 1) and/or enolase-alpha.^51^

Using morphological observation, measurements of LDH release and caspase 3/7-activation, the use of inactivated ARP and the control peptide Scr, in conjunction with Nec-1, NSA and a pan-caspase inhibitor, clearly link AChE-R to regulated necrosis of human GCs, *in vitro*. Importantly, the detection of p-MLKL in ovarian sections implicate that necroptosis occurs *in vivo* in the primate ovary, in addition to the well-examined apoptotic cell death. Regulated necrosis (necroptosis) is being implicated in human diseases.^1, 5^ Our results indicate that necroptosis is an unrecognized form of cell death of human ovarian cells and thus a part of ovarian physiology, namely follicular atresia and luteolysis. This is in line with reported necrotic cell death in Drosophila ovary^52^ and necrosis of GCs in follicles of mammalian species.^17^

We do not know how AChE-R/ARP can induce necroptosis in GCs. Based on previous studies, RACK1 and enolase^51^ may be involved, as may be TLRs or other death receptors.^53, 54^ GCs express TLR4,^55^ but neither lipopolysaccharide, a ligand of TLR4, nor TNFalpha, did induce necroptotic events in GCs (Supplementary Data). Levels of reactive oxygen species (ROS)^12^ also did not change at least during a 5-h time period of observation after addition of ARP (see Supplementary Data). AChE-R has been called a stress form of AChE, as its expression is increased upon oxidative stress.^46, 56^ It is under the control of microRNA (miRNA)-132^57^ that was elevated in exosomes of FF of growing bovine follicles *versus* fully grown follicles.^58^ Ample evidence for oxidative stress and ROS in follicles and human GCs has also been provided,^24^ and our unpublished data revealed the presence of miRNA in human GCs. Details remain to be studied.

Cultured human GCs resemble not only both GCs from the large preovulatory follicle but also luteinizing GCs from the corpus luteum. In both, the follicle and in the corpus luteum, cell death events are crucial for ovarian physiology. Atresia of many ovarian follicles allows the selection of a few ovulatory ones. Regression of the corpus luteum is necessary to allow initiation of a new ovarian cycle. It is thought that apoptosis is a major mechanism in luteal regression, that is, luteolysis.^17, 59^ Indeed, in contrast to follicular atresia, apoptosis has been the only described form of cell death in this ovarian compartment to date.^59, 60^ In follicles and the corpus luteum, a cholinergic system has been implicated in local trophic actions.^29, 61^ Our cellular studies in GCs further support and extend this view, by showing that AChE variants are important parts of this ovarian system and via their enzymatic properties degrade ACh. However, the ovarian cholinergic system has yet an additional component: non-enzymatically acting AChE-R, which induces necroptosis and thus complements the cholinergic system of the ovary in an unexpected way.

What are the possible implications of these new insights? We believe that both ovarian AChE and necroptosis could be potential targets for pharmacological intervention. For example, AChE-R levels in the human circulation increase with age^62^ and the inhibition of cholinesterases is a widely used approach in case of human Alzheimer's disease.^63^ Could such an approach also alter human ovarian functions? Could specifically the trophic actions of ACh be enhanced and would this reduce follicular atresia and/or increase the life span of the corpus luteum? And is necroptosis occurring in GCs/luteal cells, induced by AChE-R and presumably other factors, ‘treatable' with Nec-1 and NSA? This option now opens up and may be explored further.^5^ Follicular atresia, driven by apoptotic and presumably necroptotic cell death, ultimately leads to the depletion of the ovarian follicle pool and to menopause in women.^13, 14, 23^ If the cholinergic system and necroptosis do contribute significantly, it may be possible to use AChE inhibitors and/or necroptosis inhibitors to interfere with the depletion of the ovarian follicle pool during ageing and thus possibly delay menopause.

## Materials and Methods

### Human GC isolation, culture and treatment

Human GCs were derived from FF aspirates of IVF patients stimulated according to routine protocols.^19, 24, 64, 65, 66^ The local ethics committee of the University of Munich (Ludwig-Maximilian-University (LMU)) approved of the project and the use of human samples (project 323-05). Patients gave their written agreement and samples were anonymized. Aspirates from two to five patients were pooled for GCs preparation, and GCs were purified according to a method described.^67^ It involves a cell strainer (40 *μ*m; BD, Franklin Lakes, NJ, USA) for filtration of the aspirates. GCs, which remained in cell strainer, were retrieved by washing with Dulbecco's modified Eagle's medium (DMEM)/Ham's F12 Medium (PAA, Cölbe, Germany). The filtrate was centrifuged and the supernatant (i.e., cell-free FF) was frozen at −20 °C until further use. Remaining cell aggregates in the acquired cell suspension were suspended mechanically by using a 0.9-mm cannula. Washed cells were re-suspended and cultured in DMEM/Ham's F12 medium supplemented with penicillin (100 U/ml), streptomycin (100 *μ*g/ml) and 10% FCS (all from PAA).^19, 64, 65, 66^ Primary GCs were cultured for up to 6 days. Except for studies done immediately after isolation (day 0), cells were rinsed on day 1 of culture with fresh medium to remove non-adherent and dead cells. For all experiments, DMEM/Ham's F12 medium without supplements was used, except for confluence studies, where 5% FCS was added to the medium. ACh (Life Technologies, Carlsbad, CA, USA), AChE-R peptide ARP (1-GMQGPAGSGWEEGSGSPPGVTPLFSP-26^42^), a nonsense scrambled peptide (Scr; both synthesized at the GeneCenter, LMU Munich), atropine (Sigma-Aldrich, St Louis, MO, USA), HupA (Sigma-Aldrich), Nec-1 (Santa Cruz Biotechnology, Dallas, TX, USA), NSA (Tocris Bioscience, Bristol, UK) and Z-VAD-FMK (R&D Systems, Minneapolis, MN, USA) were used in several experiments.

### Confluence measurement

GCs were cultured up to 4 days and confluence of GCs was monitored for 24 h by taking time-lapse pictures every 10 min using a live cell analyzer (Peqlab, Erlangen, Germany). For each stimulation protocol, respective control cells were observed simultaneously. The software of the live cell analyzer determined confluence values.

### Ellman assay

This assay is based on a previously described method^68, 69^ with some alterations. 5,5-Dithiobis-2-nitrobenzoic acid (final concentration 0.6 mM), acetylthiocholine iodide (final concentration 1.5 mM), BW284c51 (final concentration 0.1 mM), tetra-isopropyl pyrophosphoramide (final concentration 0.1 mM) and AChE from *Electrophorus electricus* were purchased from Sigma-Aldrich. BW284c51 was used for AChE inhibition and tetra-isopropyl pyrophosphoramide for BChE inhibition. The assay was performed in 96-well plates with a total volume of 250 *μ*l. Change in absorbance at 405 nm was measured in a microplate reader (BMG labtech, Ortenberg, Germany) for 8 min. FFs were used in a final dilution of 1/320. Lysates of GCs cells cultured in serum-free medium were frozen at −20 °C, thawed and washed two times in Ellman buffer and used in a 1/8 dilution for activity measurements. Absolute activity values were determined by comparing with an AChE standard (*Electrophorus electricus*).

### Western blotting

Western blotting^24^ was performed with a goat antiserum raised against all human AChE variants (Santa Cruz Biotechnology) and the corresponding blocking peptide (Santa Cruz Biotechnology). In addition, a rabbit antiserum raised against the human AChE-R C-terminus was used.^41^ For detection of necroptosis-related proteins, we used antiserum against RIPK1 (Sigma-Aldrich), and monoclonal antibodies against RIPK3 (Sigma-Aldrich), against MLKL (Sigma-Aldrich) and against p-MLKL (Abcam, Cambridge, UK). Densities of the bands were evaluated using ImageJ (National Institutes of Health, Bethesda, MD, USA) as described.^24^

### Reverse transcription-PCR

Total RNA of cultured cells was isolated using the RNeasy Mini Kit (Qiagen, Hilden, Germany). Reverse transcription was performed with 400 ng RNA using random hexamer primers and Superscript II (Life Technologies). RT-PCR was arranged with different oligomer primers^70^ (Table 1). PCR products were analyzed by using agarose gel electrophoresis with ethidium bromide or Midori Green (Nippon Genetics Europe GmbH, Düren, Germany) staining. All products were confirmed by sequencing.^24^

### Immunohistochemistry

Sections of human ovaries derived from a local collection at Anatomy III, Cell Biology (Munich, Germany) and sections of ovaries from rhesus macaques (*Macaca mulatta*, age 5–6 years), derived from the Oregon National Primate Research Center (Beaverton, OR, USA), were used for immunohistochemistry. The collection of monkey tissues had been approved by the Oregon National Primate Research Center Institutional Animal Care and Use Committee. Immunohistochemistry was performed with the same antisera used for western blotting. Tissue samples and immunohistochemistry were described previously.^24, 66^ In brief, after removal of paraffin, antigen retrieval and blocking of endogeous peroxidase activity, the tissue was incubated in 5% appropriate serum, diluted in phosphate-buffered saline. Antiserum incubation was done overnight at 4 °C. The antiserum against all AChE variants was diluted 1 : 100, the antiserum against AChE-R 1 : 200 and the antibody against p-MLKL 1 : 50. Incubation with a biotinylated secondary antibody (1 : 500 dilution; Dianova, Hamburg, Germany) for 2 h at room temperature followed. A Vectastain ABC Kit (Vector Laboratories, Burlingame, CA, USA) and a 3,3′-diaminobenzidine tablet set (Sigma-Aldrich) were used for the final staining procedure. Slides were covered by using Entellan (Merck Millipore, Billerica, MA, USA). Controls included incubation with serum or IgG instead of the first antiserum or antibody. In case of AChE, incubation with antigen-peptide preadsorbed antiserum was employed.

### Live cell imaging

GCs were seeded in dishes with glass bottom (Ibidi, Munich, Germany) and observed on a light microscope (Carl Zeiss, Oberkochen, Germany) for 24 h after stimulation in a heated incubation chamber with constant humidity and CO_2_ concentration (Ibidi, Munich, Germany). A total of 100–200 cells were monitored in each experiment. Pictures were taken with a digital microscope camera (Jenoptik, Jena, Germany) and analyzed with the Micro-Manager software (www.micro-manager.org). By counting cell number at 0 h and cell number of dead cells at 24 h, the percentage of cell death was determined.

### LDH assay

Isolated GCs were seeded in 96-well plates for analyzing cytotoxicity of stimulants with a LDH cytotoxicity assay kit (Thermo Scientific, Waltham, MA, USA). This assay measures LDH in the cell supernatant as an indicator of damaged cell membranes. Absorbance after 5-h stimulation (between day 2 and 5 of culture) was measured at 492 nm and 690 nm (background) in triplicates with a microplate reader (BMG labtech).

### Caspase assay

Caspase 3/7 activity in GCs on 96-well plates was determined by using a caspase-glo assay system (Promega, Fitchburg, WI, USA). Luminescence after 5-h stimulation (between day 3 and day 5 of culture) was measured in triplicates on a microplate reader (BMG labtech).

### Statistics

Statistical analyses were done using Prism 5 (GraphPad Software, San Diego, CA, USA). A one-way ANOVA followed by the Newman–Keuls post-test (*P*<0.05) was performed for confluence measurements and results of cell imaging. Activity measurements were analyzed by column statistics. For caspase 3/7 assay and LDH assays with ARP stimulation, a repeated-measures ANOVA, followed by the Newman–Keuls post-test (*P*<0.05) was used. For the LDH assays with Nec-1 and NSA compared with control only, an unpaired *t*-test was performed (*P*<0.05).

## Figures and Tables

**Table 1:** Oligonucleotide primers used in RT-PCR

**Target mRNA**		**Primer sequence (5'→3')**	**Accession no.**
AChE-E	F	CGGGTCTACGCCTACGTCTTTGAACACCGTGCTTC	NM_015831.2
	R	ATGGGTGAAGCCTGGGCAGGTG	
AChE-R	F	CCCCTGGACCCCTCTCGAAAC	NM_015831.2
	R	ACCTGGCGGGCTCCCACTC	NC_018918.2
AChE-S	F	CGGGTCTACGCCTACGTCTTTGAACACCGTGCTTC	NM_000665.3
	R	CACAGGTCTGAGCAGCGATCCTGCTTGCTG	

Abbreviations: F, forward; R, reverse

## Abbreviations

ACh: acetylcholine
AChE: acetylcholinesterase
AChE-E: erythrocyte AChE variant
AChE-R: readthrough AChE variant
AChE-S: synaptic AChE variant
ARP: specific AChE-R C-terminal peptide
ARPin: heat-inactivated ARP
BChE: butyrylcholinesterase
DMEM: Dulbecco's modified Eagle's medium
DMSO: dimethyl sulfoxide
FCS: fetal calf serum
FF: follicular fluid
GC: granulosa cell
HupA: huperzine A
IgG: immunoglobulin G
IVF: *in vitro* fertilization
LDH: lactate dehydrogenase
LMU: Ludwig-Maximilian-University
miRNA: microRNA
MLKL: mixed lineage kinase domain-like protein
N-AChE: N-terminally extended AChE
Nec-1: necrostatin-1
NSA: necrosulfonamide
p-MLKL: phosphorylated MLKL
RACK: receptor of activated C kinase
RIPK: receptor-interacting serine/threonine-protein kinase
ROS: reactive oxygen species
RT-PCR: reverse transcription PCR
Scr: scrambled control peptide
TC: theca cell
TLR: Toll-like receptor
TNF: tumor necrosis factor
