# Supplementary material for: Readthrough acetylcholinesterase (AChE-R) and regulated necrosis: pharmacological targets for the regulation of ovarian functions?
Source: Cell Death Dis. 2015 Mar 12;6(3):e1685–. doi: 10.1038/cddis.2015.51 (PMC4385929; doi:10.1038/cddis.2015.51)
Supplement: Supplementary Information [file cddis201551x1.docx]

**Supplemental Data**

**Supplemental Fig. 1.** Confluence and ROS measurements, live cell imaging and RT-PCR results. **A,** TNFα (10 ng/ml) has no effect on GC confluence compared to control group during 24 h. Values are normalized to control values and given as mean ± SEM of n = 3 independent preparations of cells from two to five patients each. **B,** LPS (10 µg/ml) stimulation has no effect on confluence of cultured GCs compared to control group during 24 h. Values are normalized to control values and given as mean ± SEM of n = 3 independent preparations of cells from two to five patients each. **C,** Treatment of GCs with ARP (50 ng/ml) for 5 h does not induce the generation of ROS, monitored by the increase in the DCF-fluorescence intensities^1^. H_2_O_2_ (1 mM) was used as positive control. Values of each reading point are normalized to control values and given as mean ± SEM of n = 6 independent preparations of cells from two to five patients each. **D,** Observed cell death rate increases significantly (p < 0.05; ANOVA, Newman-Keuls) after 24 h ARP treatment compared to control groups. Values are the mean ± SEM of n = 5 (control, ARP inactivated) and n = 10 (ARP, Scr) independent preparations of cells from two to five patients each. **E,** RIPK1 and MLKL mRNA is detectable in human GCs. RNA and water controls are negative. For each primer one RT-PCR product was confirmed by sequencing. **F,** Densitrometric analysis of the p-MLKL/MLKL Western blot experiment. Total and phosphorylated MLKL levels were determined [arbitrary units (AU] and p-MLKL levels were normalized to total MLKL levels. Evaluation of the data shows a tendency to an increase of MLKL phosphorylation after ARP treatment compared to control group. Values are normalized to control values and given as mean ± SEM of n = 3 independent preparations of cells from two to five patients each.

**Reference:**

1. Saller S, Kunz L, Berg D, Berg U, Lara H, Urra J*, et al.* Dopamine in human follicular fluid is associated with cellular uptake and metabolism-dependent generation of reactive oxygen species in granulosa cells: implications for physiology and pathology. *Hum Reprod* 2014; **29**(3)**:** 555-567.
